# Trend of prostate cancer in Fars Province, Southern Iran, 2001-2007

**Published:** 2010

**Authors:** Mahin Farahmand, Farnaz Khademolhosseini, Davood Mehrabani

**Affiliations:** a Non Communicable Disease Division, Office of Vice Chancellor for Health Affairs, Shiraz University of Medical Sciences, Shiraz, Iran,; b Gastroenterohepatology Research Center, Shiraz University of Medical Sciences, Shiraz, Iran,; c Iranian Hospital, Dubai, UAE

In the United States, the estimates for prostate cancer in 2008 included over 186000 new cases and about 28700 deaths.^1^ It is one of the two most common malignancies among men in Gulf Cooperation Council countries.^2^ In Iran, prostate cancer has an incidence of 9.6 per 100,000, ranging from 3.2 to 16.0 per 100,000 in different geographical settings.^3^ This study aims to determine the trend of prostate cancer in Fars Province, Southern Iran.

On a population based cancer registry, all cases of prostate cancer diagnosed in Fars Province, southern Iran from April 2001 to April 2007 were enrolled. Data were collected from all private and public pathology centers as well as forensic medicine laboratories and death/birth registries in the province. After coding the data according to ICD-O, they were classified and recorded in a computer database. Demographic data including age, residential area and morphology of the tumor were recorded.

**Figure 1:**
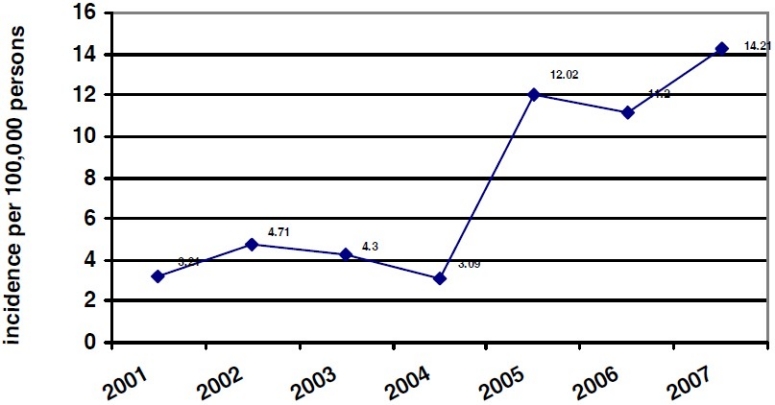
Incidence of prostate cancer in Fars Province, Southern Iran during 2001-2007

A total of 1102 cancer patients were registered. Out of these patients, 134 cases (12.5%) were resided outside Fars Province and the residential address of 110 cases (9.98%) was not found in the patient’s records. The incidence of prostate cancer had an increasing trend from 3.24 cases per 100,000 in 2001 to 14.21 cases per 100,000 in 2007 (Figure 1). The actual number of diagnosed prostate cancer cases increased from 64 in 2001 to 285 in 2007. The incidence rate was constant through 2001 to 2004 with a dramatic increase from 2004 to 2007. Improvement in surveillance systems and cancer registration may partly explain the increase in incidence.

This result on incidence is similar to that of Mousavi, who reported the incidence rate from 3.2 to 16.0 per 100,000 in different regions.^3^ The increasing trend in Fars Province is identical to Matsuda et al report in Asia showing an increasing trend except in India, where there was no obvious increase and decrease.^4^

**Figure 2:**
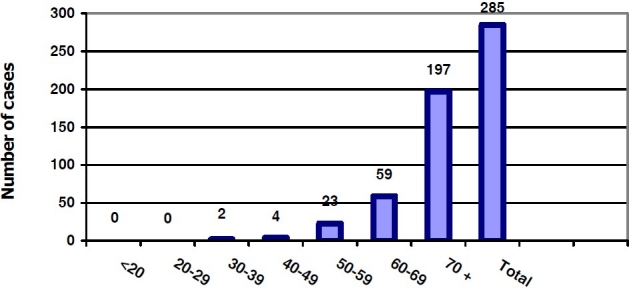
Frequency distribution of prostate cancer according to age in Fars Province, Southern Iran in 2007

Figure 2 shows that there is no diagnosed case of prostate cancer under the age of 30, and the rate increases with age. These results are similar to those of Mehrabani et al study in Fars with no cases of prostate cancer under the age of 25, and with an age increasing trend,^5^ and to that of Parkin et al who reported that 75% of prostate cancers were in age group of ≥ 65 years.^6^

**Figure 3:**
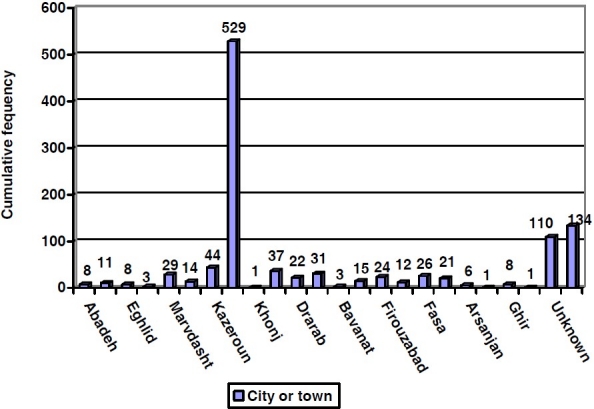
Cumulative frequency of prostate cancer in different cities of Fars Province, Southern Iran during 2001-2007

The cumulative frequency of diagnosed prostate cancer in different cities of Fars Province is depicted in figure 3. A total of 529 patients were from Shiraz and then Kazeroun, Lar, and Mamasani had the highest frequencies. The relative frequency was lowest in 2001 (5.4%) and increased to its highest value (10.04%) in 2005, but since then it declined and reached 8.9% in 2007. Morphology of tumor for all prostate cancer was consistently found to be adenocarcinoma.

These findings should be taken into account in future health and medical planning.
